# DYNAMO-HIA–A Dynamic Modeling Tool for Generic Health Impact Assessments

**DOI:** 10.1371/journal.pone.0033317

**Published:** 2012-05-10

**Authors:** Stefan K. Lhachimi, Wilma J. Nusselder, Henriette A. Smit, Pieter van Baal, Paolo Baili, Kathleen Bennett, Esteve Fernández, Margarete C. Kulik, Tim Lobstein, Joceline Pomerleau, Johan P. Mackenbach, Hendriek C. Boshuizen

**Affiliations:** 1 Department of Public Health, Erasmus MC, University Medical Center Rotterdam, Rotterdam, The Netherlands; 2 Department of Statistics and Mathematical Modeling, National Institute for Public Health and the Environment (RIVM), Bilthoven, The Netherlands; 3 Julius Center for Health Sciences and Primary Care, University Medical Center Utrecht, Utrecht, The Netherlands; 4 iBMG/iMTA, Erasmus University Rotterdam, Rotterdam, The Netherlands; 5 Expertise Centre for Methodology and Information Services, National Institute for Public Health and the Environment (RIVM), Bilthoven, The Netherlands; 6 Fondazione IRCCS “Istituto Nazionale dei Tumori”, Descriptive Studies and Health Planning Unit, Milan, Italy; 7 Department of Pharmacology & Therapeutics, Trinity Centre for Health Sciences, St James’s Hospital, Dublin, Ireland; 8 Tobacco Control Unit, Cancer Control and Prevention Programme, Institut Català d’Oncologia-ICO, L’Hospitalet de Llobregat, Barcelona, Spain; 9 Cancer Control and Prevention Group, Institut d’Investigació Biomèdica de Bellvitge-IDIBELL, L’Hospitalet de Llobregat, Barcelona, Spain; 10 Department of Clinical Sciences, School of Medicine, Universitat de Barcelona, L’Hospitalet del Llobregat, Barcelona, Spain; 11 Center for Prevention and Health Services Research, National Institute for Public Health and the Environment (RIVM), Bilthoven, The Netherlands; 12 Director of Policy and Programmes,IASO - the International Association for the Study of Obesity IOTF - the International Obesity TaskForce, London, United Kingdom; 13 Department of Biometrics, Wageningen University, Wageningen, The Netherlands; McGill University Health Centre, McGill University, Canada

## Abstract

**Background:**

Currently, no standard tool is publicly available that allows researchers or policy-makers to quantify the impact of policies using epidemiological evidence within the causal framework of Health Impact Assessment (HIA). A standard tool should comply with three technical criteria (real-life population, dynamic projection, explicit risk-factor states) and three usability criteria (modest data requirements, rich model output, generally accessible) to be useful in the applied setting of HIA. With DYNAMO-HIA (Dynamic Modeling for Health Impact Assessment), we introduce such a generic software tool specifically designed to facilitate quantification in the assessment of the health impacts of policies.

**Methods and Results:**

DYNAMO-HIA quantifies the impact of user-specified risk-factor changes on multiple diseases and in turn on overall population health, comparing one reference scenario with one or more intervention scenarios. The Markov-based modeling approach allows for explicit risk-factor states and simulation of a real-life population. A built-in parameter estimation module ensures that only standard population-level epidemiological evidence is required, i.e. data on incidence, prevalence, relative risks, and mortality. DYNAMO-HIA provides a rich output of summary measures – e.g. life expectancy and disease-free life expectancy – and detailed data – e.g. prevalences and mortality/survival rates – by age, sex, and risk-factor status over time. DYNAMO-HIA is controlled via a graphical user interface and is publicly available from the internet, ensuring general accessibility. We illustrate the use of DYNAMO-HIA with two example applications: a policy causing an overall increase in alcohol consumption and quantifying the disease-burden of smoking.

**Conclusion:**

By combining modest data needs with general accessibility and user friendliness within the causal framework of HIA, DYNAMO-HIA is a potential standard tool for health impact assessment based on epidemiologic evidence.

## Introduction

Health Impact Assessment (HIA) is a combination of procedures, methods, and tools that judges the effect of (intended) programs, projects, or policies on overall population health and the distributional effects within a population [1]. The rationale behind HIA is that many risk-factors for chronic diseases are affected by policy measures outside the realm of health policy (e.g. transportation, food, or urban planning). Health impact assessments have been carried out at all governmental levels (e.g. local [2], regional [3], national [4], and supranational [5]). The number of HIAs is likely to rise due to increased institutional adoption [6] and political will, in particular at EU level [7]. Currently, there is a diversity of approaches to the quantification of policy interventions [8]. However, for the quantification step in HIA, a generic modeling tool – i.e. allowing for various and multiple chronic diseases and arbitrary risk-factors – that takes into account the standard causal pathway assumed in HIA has been lacking [9].

The standard HIA causal pathway assumes that a policy intervention leads to a change in risk-factor prevalence which in turn leads to changes in disease incidence and disease-related mortality [10]. The two objectives of HIA – to predict future consequences of implementing different options and to inform decision makers in choosing between options [11] – address the technical core of quantification (*predict*) as well as the context (*inform*) in which an HIA takes place. Hence, a potential standard tool should aim for technical accuracy in the prediction of the effects of interventions on population health, and yet be effective in the applied setting of an HIA, where time and resources are scarce. These objectives were operationalized into six criteria that a generic model should fulfill to be useful as a standard tool [9]. The first three criteria (*real-life population*, *dynamic projection*, and *explicit risk-factor states*) ensure that the model structure is sufficiently accurate in modeling changes in risk-factor exposure over time in a real-life population in a transparent way. The last three criteria (*modest data requirements*, *rich model output*, and *generally accessible*) ensure a wide usability by accounting for the constraints of a decision-making process.

This article proposes a software – DYNAMO-HIA (DYNamic MOdeling for Health Impact Assessment) –as a standard tool for the quantification of user-specified policy interventions within the HIA-framework.

## Materials and Methods

### Implementation of Requirements for a Standard Tool

We designed DYNAMO-HIA to satisfy the six criteria that a generic standard tool for HIA should fulfill. DYNAMO-HIA models a closed *real life population*, i.e. stratified by sex and age in 1 year age categories up to the age of 95 without migration (including the expected number of newborns). The model is *dynamic* in 1-year time steps and projects reference and (several) intervention scenario(s) over time. DYNAMO-HIA has *explicit risk-factor states*, i.e. at every time step of the simulation each simulated individual is classified into a specific risk-factor category. This ensures an accurate, unbiased estimation and increases the transparency of the simulation and the resulting output data.

DYNAMO-HIA has a parameter estimation module, mostly using methods taken from the Chronic Disease Model of Dutch National Institute for Public Health (RIVM-CDM) [12], *reducing data needs* substantially. Incidence and prevalence of a disease are only needed at the population level, i.e. specified by age and sex and not by each risk-factor state. The module back-calculates the risk-factor specific values using the relative risk from each risk-factors state on diseases. The user can inspect these intermediate results when desired, thus improving transparency. DYNAMO-HIA provides *rich simulation output* available in three forms: (1) raw output data, allowing detailed analysis by age, sex, and risk-factor status. This raw data give either the cohort disease life table for every simulated cohort or the period data for every simulated year; (2) several dynamic plots, e.g. population pyramids or survival rates, based on the data that contrast key information between the reference scenario and the intervention scenario; (3) a range of summary outcome measures, e.g. cohort-, period-, or disease-free life expectancy. The graphical user interface allows *general accessibility*; no programming or other advanced computing skills are required.

### Model Core

**Figure 1:**
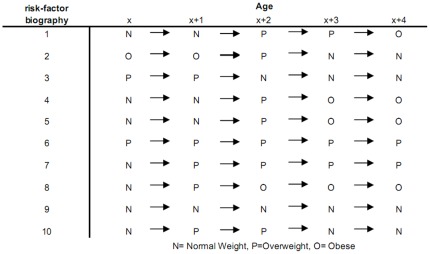
Example of risk-factor biographies for a risk-factor with three categories. DYNAMO-HIA simulates individuals and projects their risk-factor biographies. The risk-factor status is being updated in one-year increments, given age- and sex-specific transition probabilities. The age- and sex-specific risk-factor status determines the relative risk of a person to contract a disease or to die. DYNAMO-HIA allows one risk-factor per scenario. This risk-factor can be either categorical (up to ten categories), duration dependent (up to ten categories, of which one is duration dependent, i.e. the risk on disease in this category depends on how long a person is in the category), or a continuous distribution (normal or log-normal, specified by entering mean, standard deviation, and, in the case of the log-normal, skewness).

**Figure 2:**
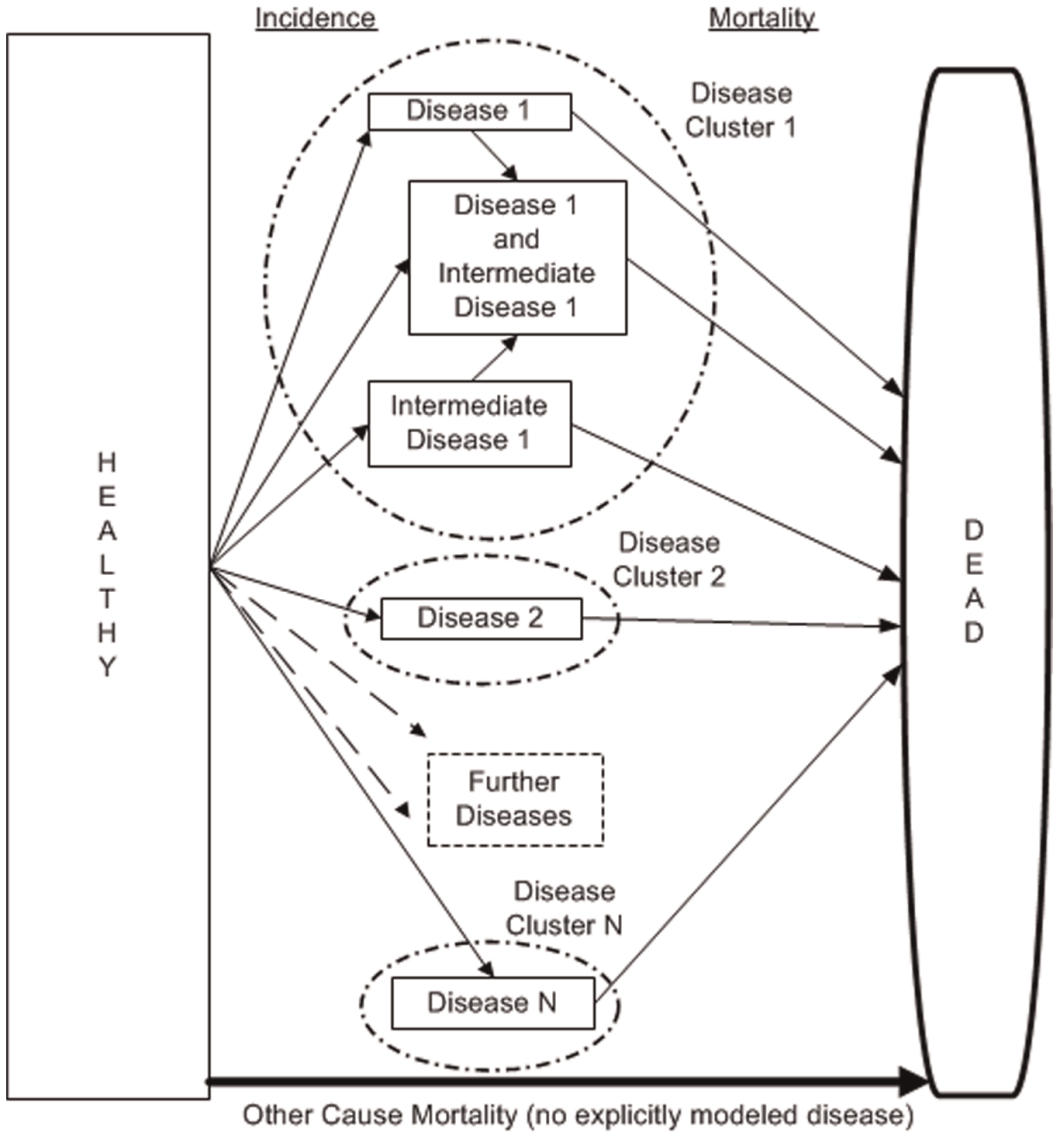
Stylized structure of disease life table. The disease life tables contain disease clusters. Each disease cluster consists of one or more diseases. Within disease clusters, intermediate diseases – that is, a disease that increase the risk of getting another disease – can be specified (e.g. having diabetes increases the risk of getting IHD). All diseases are chronic diseases, i.e. excess mortality depends on age and sex and not on time since onset of disease. However, acutely fatal and/or cured fraction can be specified for diseases. The disease life table assumes independence between disease clusters. The user can freely specify the relative risks from risk-factor to disease, from risk-factor to death, and from intermediate disease to other diseases.

**Figure 3:**
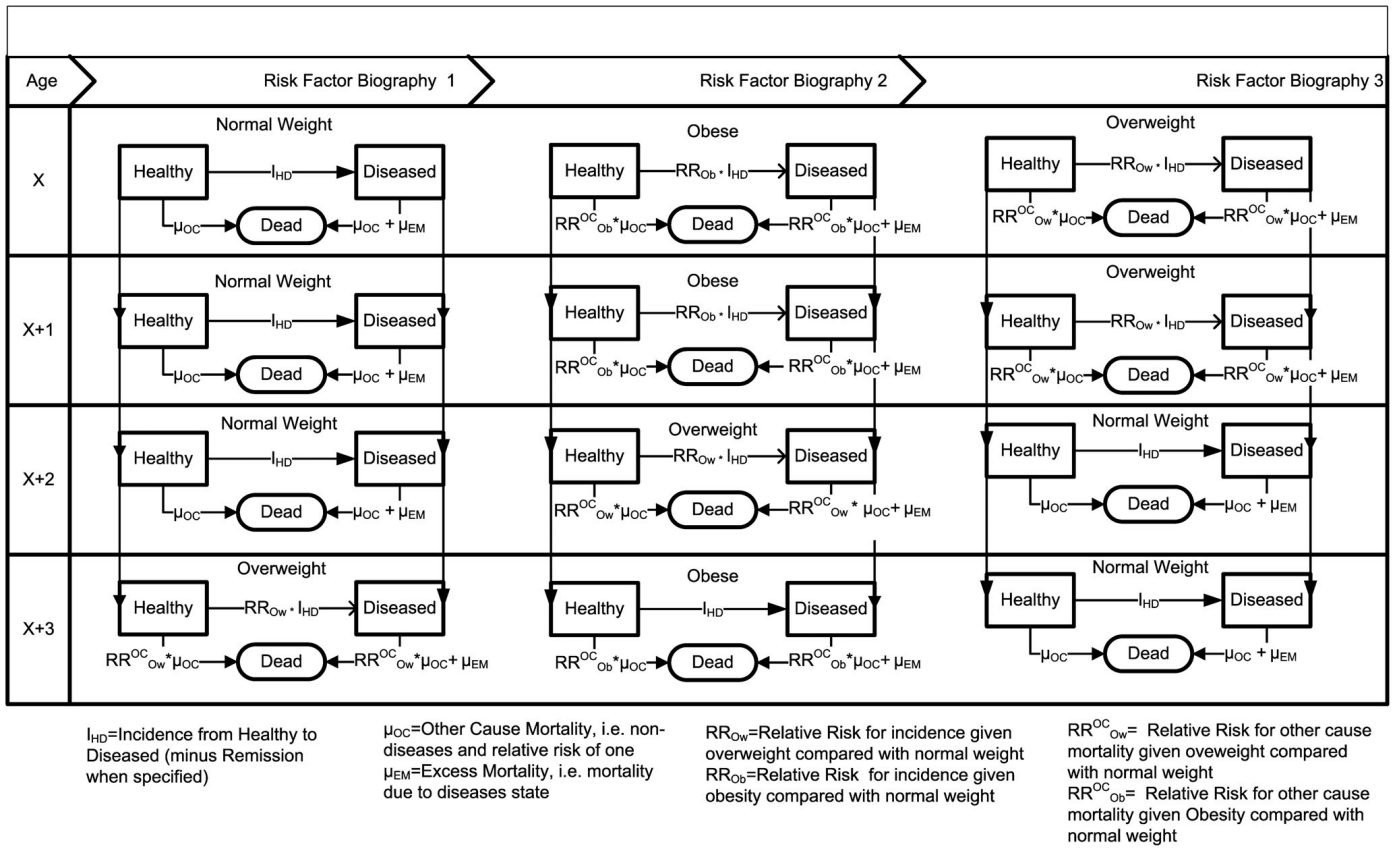
Stylized cohort life tables (with only one disease, three different biographies, and five time steps). For every risk-factor biography, a disease life table is constructed. Diseases incidence, i.e. transition from a healthy to a diseased status, equals the baseline incidence – that is, the incidence when in a risk-factor class with a relative risk of one for the specific age- and sex-category – times the relative risk due to the given risk-factor and diseases status (in the case of an intermediate disease). The transition from healthy to dead equals the baseline other-cause mortality of the healthy, i.e. age- and sex-specific total mortality rate minus the excess mortality rate of the diseases included in the disease life table, multiplied by the relative risk due to the given risk-factor status on other-cause mortality. The transition from diseased to dead equals the sum of the excess mortality of the disease (given each age and sex) and the baseline other-cause mortality of the healthy, multiplied by the relative risk in the given risk-factor status. Remission is not explicitly modeled, but for diseases with cured fraction the excess mortality is zero in a “cured”, i.e. user-specified, fraction. Partly acutely fatal diseases, i.e. diseases with very high mortality immediately after contracting the disease while for those who survive this critical period the excess mortality only depends on age and sex, are modeled by specifying the fraction of the incidence cases that die immediately.

**Figure 4:**
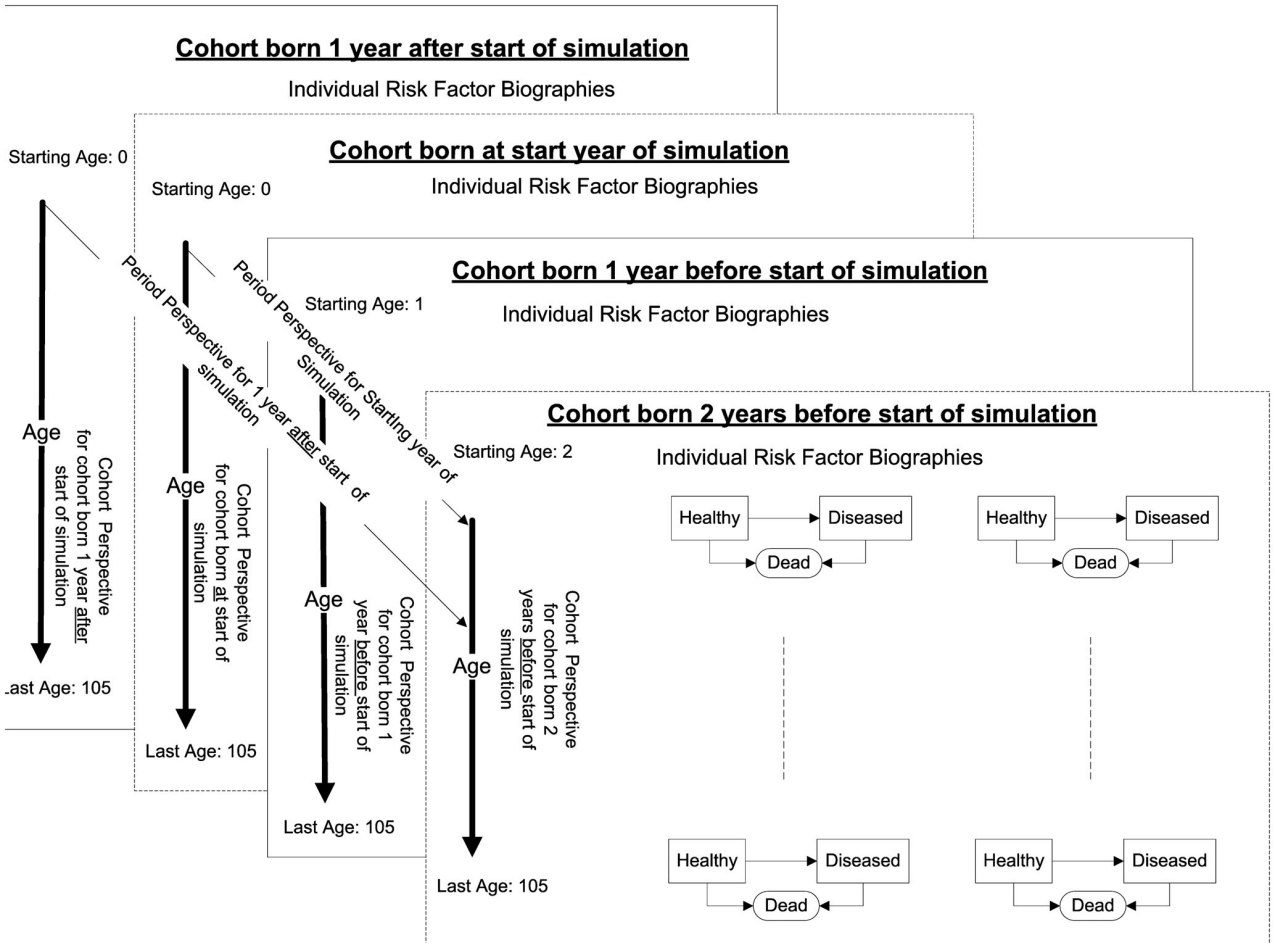
Schematic overview of the dimension of a multi-cohort, multistate-life table. Each plane is a distinct cohort with varying starting ages for cohorts already existing at the starting year of the simulation and starting age zero for cohorts born during the simulation run. The cohort life tables, consisting of the set of individual risk-factor biographies, follow every already existing birth cohort until the cohort reaches 105 years of age. In addition, every year of the simulation a cohort of newborns is created and – after simulating individual risk-factor biographies for them – is followed through the appropriate disease life tables as well. This allows collecting health data for each cohort according to their risk-factor status (longitudinal) or the health status of the population by age, sex, and risk-factor status by each year of the simulation (cross-sectional).

DYNAMO-HIA is a Markov-type model based on a multi-state model (MSM). The change of the state depends only on current characteristics, i.e. age, sex, risk-factor status, and health status. The MSM is implemented as a *partial micro-simulation* combining a stochastic micro-simulation to project risk-factor behavior with a deterministic macro approach for the disease life table [13]. In the micro-simulation, module large numbers of distinctive risk-factor biographies are simulated: Given the age and sex-specific transition probabilities between risk-factor states, the risk-factor status of each simulated individual is updated in annual increments (see Fig. 1 for details). In the macro module, a separate disease life tables is constructed for each risk-factor biographies. These disease life tables account for competing risks and multiple morbidity [14]. The exact configuration of the disease life tables, i.e. the number and types of diseases, can be specified by the user (see Fig. 2 for details). For every risk-factor biography, the probability of disease incidence and mortality over time is calculated, accounting for the current age, risk-factor, and disease status (see Fig. 3 for details). These biography-specific life tables are calculated for each birth-cohort, i.e. all individuals that are born in the same calendar year. For example, for a cohort of newborns, risk-factor biographies are projected and subsequently disease life tables are calculated. Older cohorts, i.e. those born before the first simulation year, already have the disease prevalence as specified by the input data, which is then similarly updated. Population values are obtained by aggregating the individual biography/diseases life tables: either across cohorts at a given simulation time point to obtain period measures or along cohorts to obtain cohort specific measures (see Fig. 4 for details). The split into a micro and a macro module is done purely for computational convenience; micro- and macro-simulations yield the same result when used with the same data [15], [16]. However, time and memory requirements in macro-simulations rise exponentially when the number of simulated states increases. In contrast, micro-simulations – unlike customary multi-state life tables – do not require the a priori specification of all theoretically possible combinations of diseases/risk-factor states, but only those states that are actually occupied. However, for simulating rare events – e.g. lung cancer at young ages – micro-simulations require the simulation of large numbers of individuals, offsetting the savings in time and memory requirements.

The epidemiological model uses relative risks by risk-factor class, i.e. incidences in exposed risk-factor classes are a multiple of the incidence in non-exposed. The total mortality, i.e. population level mortality by age and sex, is being decomposed in the mortality due to the diseases included in the model and other-cause mortality. This decomposition assumes additive mortality: the total mortality rate in the population is explained as the sum of the mortality rate of the included diseases and other-cause mortality, i.e. mortality from all causes/diseases that are not explicitly included in the model.

### Modeling Policies with DYNAMO-HIA

The goal of HIA is to compare the effect of several policies/interventions on future population health, keeping the status quo as the reference scenario. Within DYNAMO-HIA, policies can be modeled in two ways (both approaches can be applied simultaneously and/or targeted at selected parts of the population only). The first approach is to define a counterfactual risk-factor prevalence that is assumed to be reached after a successful one-time, sustained intervention, e.g. a reduction in alcohol consumption caused by a tax increase or a ban on smoking in public. The approach of defining counterfactual risk-factor prevalences is akin to epidemiological methods, where total or partial eradication of a risk-factor is quantified. DYNAMO-HIA does this quantification dynamically, i.e. effects are projected over time. The second approach is to alter the transition probabilities between different risk-factor states, i.e. changing the risk-factor behavior of the population. This approach is closer to the reality of many health interventions that try to influence life style choices of individuals, e.g. halving the future number of teenagers that become obese. The specification of the transition probabilities influences greatly the future development of the risk-factor prevalence, which is always debatable. As an option, DYNAMO-HIA provides the use of net transition probabilities [17]: DYNAMO-HIA estimates internally the transition probabilities that keep the age-specific risk-factor prevalence constant, ignoring any future cohort effects.

### Illustration

To illustrate the usability of DYNAMO-HIA, we present two stylized example applications. The first illustration projects the consequences of a policy-induced increase in alcohol consumption and resembles a prospective HIA. The second illustration quantifies the changes in population health if smoking were to be eradicated and resembles a burden of disease study. In both applications, we model the effects of risk-factors on total mortality and nine diseases (ischemic heart disease, stroke, diabetes, COPD, breast-, lung, esophageal-, colorectal-, and oral-cancer) and keep the age-specific risk-factor prevalence constant over time by using net transition probabilities between risk-factor classes, i.e. ignoring any future cohort effects. Hence, the difference between the reference scenarios and the intervention scenarios depends solely on the different initial risk-factor prevalences. The data sources and the relative risk used are shown in detail in the supporting information (see Table S1, Table S2, Table S3, Table S4, and Table S5).

#### Liberalizing access to alcohol: The swedish case

**Figure 5:**
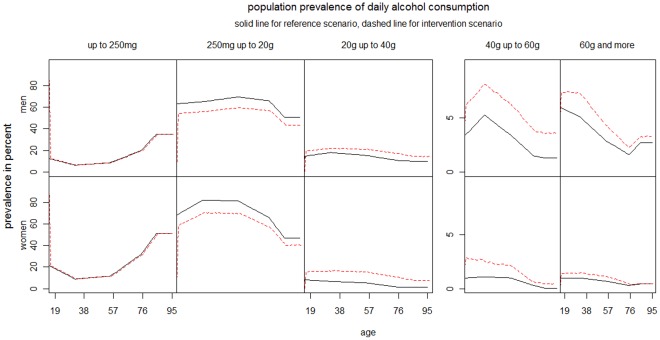
Swedish prevalence of alcohol consumption intervention scenario compared with reference scenario (Alcohol consumption is measured by five categories of daily intake of grams of pure alcohol: 0–<0.25 g/d, 0.25–<20 g/d, 20–<40 g/d, 40–<60 g/d, ≥60 g/d).

In 2004, Sweden was forced to lift her its ban on private alcohol imports [18]. Prospective studies were forecasting an increase in overall alcohol consumption and consequently a worsening of a number of alcohol-related harm indicators. In our reference scenario, we keep the alcohol consumption prevalence observed in 2002 constant during the projection period and assume a one-time change in the consumption of pure alcohol by 1 L per-capita, producing a counterfactual risk-factor prevalence for the intervention scenario as seen in Fig. 5. We project both scenarios for 25 years in the future (see Table 1).

**Table 1:** Number of disease cases and population prevalence (in percent) for example applications.

		Swedish Alcohol Example					UK Smoking Example			
		Reference Scenario		Intervention Scenario (1 L increase per capita alcohol consumption)			Reference Scenario		Intervention Scenario (All are never smokers)	
	year	numbers	%.	numbers	%	year	Numbers	%	numbers	%
*IHD*	*1*	354,747	3.9%	354,747	3.9%	1	2,183,447	3.6%	2,183,447	3.6%
	*25*	428,727	4.7%	428,026	4.6%	25	2,666,917	4.4%	2,144,465	3.5%
*Stroke*	*1*	150,271	1.7%	150,271	1.7%	1	1,002,594	1.7%	1,002,594	1.7%
	*25*	192,924	2.1%	194,616	2.1%	25	1,374,698	2.3%	1,156,176	1.9%
*Diabetes*	*1*	368,787	4.1%	368,787	4.1%	1	1,559,679	2.6%	1,559,679	2.6%
	*25*	385,216	4.2%	391,793	4.3%	25	1,850,392	3.1%	1,999,847	3.2%
*Lung*	*1*	4,613	0.1%	4,613	0.1%	1	82,082	0.1%	82,082	0.1%
*Cancer*	*25*	5,753	0.1%	5,750	0.1%	25	107,393	0.2%	20,334	0.0%
*Oral*	*1*	9,377	0.1%	9,377	0.1%	1	55,804	0.1%	55,804	0.1%
*Cancer*	*25*	11,738	0.1%	12,495	0.1%	25	70,949	0.1%	33,013	0.1%
*Esophageal*	*1*	971	0.0%	971	0.0%	1	11,231	0.0%	11,231	0.0%
*Cancer*	*25*	1,241	0.0%	1,300	0.0%	25	14,535	0.0%	5,324	0.0%
*Colorectal*	1	36,415	0.4%	36,415	0.4%	1	248,380	0.4%	248,380	0.4%
*Cancer*	25	47,775	0.5%	48,062	0.5%	25	329,116	0.5%	370,596	0.6%
*Breast*	1	90,444	1.0%	90,444	1.0%	1	543,738	0.9%	543,738	0.9%
*Cancer*	25	108,854	1.2%	110,661	1.2%	25	670,013	1.1%	705,919	1.1%
*COPD*	1	105,052	1.2%	105,052	1.2%	1	525,247	0.9%	525,247	0.9%
	25	131,118	1.4%	130,850	1.4%	25	1,016,422	1.7%	278,194	0.4%
*With at least*	1	918,921	10.2%	918,921	10.2%	1	5,148,112	8.6%	5,148,112	8.6%
*one disease*	25	1,081,720	11.7%	1,088,547	11.8%	25	6,726,107	11.1%	5,792,303	9.4%
*Size of*	1	9,002,148		9,002,148		1	59,987,010		59,987,010	
*total population*	*25*	9,210,437		9,206,131		25	60,416,567		61,929,848	

*For data sources see Table S1, Table S2, Table S3, Table S4, Table S5.*

The annual excess number of deaths due to increased alcohol consumption is on average approx. 170 deaths, accruing to some 4,300 additional deaths over the 25 year period. This projected difference in overall population mortality also reflects all other effects a risk-factor has on other-cause mortality, accounting for not included diseases and – more salient in the case of alcohol – injuries/accidents via the relative risk of a risk-factor on total mortality. This absolute number is rather small compared to the overall population of some 9 million; hence, the effect on total life expectancy and, similarly, the overall difference in disease-free life expectancies between the reference and the intervention scenario are negligible.

Alcohol intake has a pronounced effect on a number of the diseases projected in the model. In projection year 25, the biggest difference in absolute cases is for diabetes with approx. 6,600 more cases, followed by stroke with an excess prevalence of approx. 1,700 cases. Ischemic heart disease, the most prevalent of the included diseases, is overall less affected by the change in alcohol intake. The population prevalence differs only marginally over the simulation period, but still accounts for approx. 700 additional cases; this is partly caused by the beneficial effect of moderate drinking by some age groups (see Table S3). From the five included cancers, the increase in breast cancer is the most notable: in projection year 25, the excess prevalence is approx. 1,800 cases in the intervention scenario. For the other cancers, the increase in prevalence cases is relatively minor: for oral cancer approx. 750, for colorectal cancer approx. 280, and for esophageal cancer approx. 60 additional cases in the counterfactual scenario. COPD shows a slight decrease in absolute numbers, although it is not causally related to alcohol intake. This is due the higher number of deaths, thus there are less persons alive to contract this disease.

#### Total elimination of smoking: A projection with UK data

Smoking is a major public health concern. This illustration quantifies the gain in population health obtainable if an entire population would consisted of never smokers compared to a real life population that keeps the currently observed smoking behavior unchanged. Smoking is measured in three categories (never-, former-, current-smoker). The data for this illustration are from the UK and projected 25 years into the future (see Table 1 and Table 2). In the counterfactual, the whole population consists of never smokers and no uptake of smoking.

**Table 2:** Period based total life expectancy and expected number of years with a disease for the UK example application.

			Women			Men		
	year	Reference Scenario	Difference between Reference and Intervention Scenario	Intervention Scenario (All are never smokers)	Reference Scenario	Difference between Reference and Intervention Scenario	Intervention Scenario (All are never smokers)	
*total life*	*1*	81.3	0.2	81.5	77.0	0.8		77.8
*expectancy*	*25*	81.0	1.8	82.8	76.9	3.1		80.0
*IHD*	*1*	3.3	0	3.3	4.3	0.2		4.5
	*25*	3.0	–0.4	2.6	3.8	–0.6		3.2
*Stroke*	*1*	1.9	0	1.9	1.8	0.1		1.9
	*25*	1.8	–0.2	1.6	1.8	–0.2		1.6
*Diabetes*	*1*	2.3	0	2.3	2.6	0.1		2.7
	*25*	2.1	0.1	2.2	2.5	0.3		2.8
*Lung*	*1*	0.1	0	0.1	0.2	0		0.2
*Cancer*	*25*	0.1	–0.1	0.0	0.2	–0.2		0.0
*Oral*	*1*	0.1	0	0.1	0.1	0		0.1
*Cancer*	*25*	0.1	–0.1	0.0	0.1	–0.1		0.0
*Esophageal*	*1*	0.0	0	0.0	0.0	0		0.0
*Cancer*	*25*	0.0	0	0.0	0.0	0		0.0
*Colorectal*	*1*	0.4	0	0.4	0.5	0		0.5
*Cancer*	*25*	0.4	0.1	0.5	0.5	0.1		0.6
*Breast*	*1*	1.7	0	1.7	n/a	n/a		n/a
*Cancer*	*25*	1.7	0.1	1.8	n/a	n/a		n/a
*COPD*	*1*	0.8	0	0.8	0.9	0.1		1.0
	*25*	1.3	–0.9	0.4	1.2	–0.9		0.3
*With at least*	*1*	8.9	–0.8	8.8	8.4	0.3		8.7
*one disease*	*25*	8.7	–0.8	7.8	8.2	–0.9		7.3

*For data sources see Table S1, Table S2, Table S3, , Table S5.*

In 25 years, the population of non-smokers is projected to have approx. 1,510,000 more individuals than a population keeping the current smoking behavior. This translates into a total life expectancy of 81.4 years for the counterfactual compared to 79.0 years for the reference scenario. This gain in life expectancy is substantially larger for men than for women: For men the difference is more than 3 life years (76.9 in the reference scenario compared to 80.0 in the intervention scenario) and women 1.8 years (81.0 compared to 82.8). The projected life expectancies clearly demonstrate that in DYNAMO-HIA no autonomous trends are assumed, e.g. a secular increase in life expectancy that one may expect over the next 25 years.

Smoking also has a causal effect on a number of diseases. The biggest reduction in the modeled diseases is for COPD. In projection year 25, the average life years lived with COPD is approx. 0.9 years less in the intervention scenario than in the reference scenario, more than halving population prevalence from 1.7% to .5%. The next biggest reduction is for IHD, with approx. half a year less expected life years with this disease; a difference in prevalence of one percentage point. Similarly, the prevalence of stroke goes down by approx. 0.4 percentage points (from 2.3% to 1.9%). The three included cancers that are related to smoking are reduced as well (lung cancer by approx 87,000 cases, esophageal cancer by approx 38,000, and oral cancer by approx 9,200 cases). However, other included diseases that are not causally related to smoking (diabetes, breast-, and colorectal cancer) increase in prevalence thanks to the larger number of surviving individuals that are now at risk of contracting those diseases.

## Discussion

Within the rapidly developing field of HIA no standard method on quantification has emerged yet [19], but three approaches predominate the field: regression based methods, quantitative risk assessment, and population health models. The regression based methods originated in econometrics and usually estimate the long term relationship between exposure (e.g. per-capita consumption) or proxy variables (e.g. tax rate on alcohol) and health outcomes of interest on an aggregate level, adjusting for further variables as suggested by (economic) theory. This approach usually takes only limited notice of underlying epidemiological mechanisms. Quantitative risk assessment, originating from (environmental) exposure assessment of toxic substances, makes explicit use of dose-response relationships derived through epidemiological studies. These approaches are usually static, i.e. do not account for changes over time in real-life populations. Population health models combine epidemiological evidence and insights on causality to dynamically quantify the effect of risk-factors on population health.

DYNAMO-HIA fills a gap among the already existing population health models that are suggested for application in HIA [9], [20]. Compared to existing models, DYNAMO-HIA strikes a balance between being sufficiently technically accurate and ensuring wide usability. Technically equal or more complex models – e.g. POHEM, ARMADA, RIVM-CDM – allow for greater flexibility in modeling but are not publicly available, and require highly specialized input data and proficiency in specialized programming languages (except ARMADA). More accessible models – e.g. PREVENT, Proportional Multi-state Life Table (MSLT), GBD – lack dynamic projection capabilities (except PREVENT and multiple cohort versions of the MSLT [21]) and do not have explicit risk-factor states. This technical simplification ignores mortality selection and may lead to biased estimates [9].

**Figure 6:**
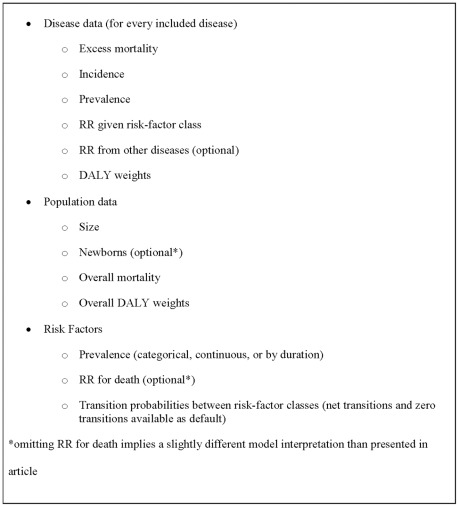
Overview of required input data (age- and sex-specific).

DYNAMO-HIA is specially designed to fit within the standard framework of HIA, synthesizing elements of already well established modeling approaches. Our approach allows for a flexible risk-factor configuration (categorical, duration dependent, continuous); generic chronic diseases as specified by the user (with intermediate diseases, partially fatal diseases, and/or diseases with a cured fraction); arbitrary specification of– age and sex-specific – relative risks; and minimal data needs by requiring only population level data (see Fig. 6). Furthermore, a mouse-driven graphic user interface allows straightforward handling of the software, i.e. no knowledge of a programming language is required. In addition to exporting the existing, partly customizable, graphs into files – e.g. detailed plots of mortality rates or prevalences of risk-factors or diseases, both over time and age-specific – most calculated data can be exported for use in separate software (e.g. Excel). These raw output data allow further analyses, such as grouping diseases into categories (e.g. IHD and stroke or all cancers), including costs, or constructing additional graphs.

DYNAMO-HIA simulates the effect of a single risk-factor on a population without migration. However, the categorical risk-factor can be used to partition the population in up to ten distinctive categories. For example, a population could be partitioned along a risk-factor – say, non-smokers and smokers – and socio-economic status – say, with and without college education – having in total four different groups to assess policies that are more successful for people with certain socio-economic status. The possibility of partitioning a population also allows quantification of the effect of an environmental hazard. In this case, for example, the population is partitioned according to their proximity to the hazard source – say, noise exposure or air pollution due to a new airport – with 5% of the total population living less than 5 km from the hazard source, 5% to 10% living less than 10 km and so on. This requires, of course, sufficient insight into which part of the population is affected and knowledge of the relative risks of the modeled exposure on the included diseases and total mortality.

A category may also represent a combination of known risk-factors: For example, smoking status and BMI – smoking/non-smoking and normal weight/overweight/obese – could be modeled by partitioning the population into six distinctive risk-factor categories. However, this would require knowledge about the relative risk of the combined risk-factor class – say, relative risk of being obese and a smoker on the included diseases and total mortality.

The overall performance of a model crucially depends on the quality of the input data. In particular for dynamic models, the epidemiological data has to be mutually consistent, otherwise projected changes in the prevalences might be caused by mismatching data and not by the changes in the risk-factors. A limitation is that an autonomous trend in the rates, e.g. annual reduction in overall mortality or disease incidence, cannot be specified. Autonomous trends are often observed for past time periods and caused by a number of factors; chief among them improved curative interventions and changed risk-factor behavior. In a risk-factor based model, however, the specification of a future autonomous trend must be net of any underlying risk-factor behavior, as this is already specified explicitly at some other place in the model. Such specific data on future trends is hardly reliably available, if at all, and would, in most cases, only modestly affect the difference between reference and intervention scenarios. Hence, an ordinal ranking of policy alternatives would be rarely affected while still revealing the most effective intervention.

In health impact assessment, three criteria are used to assess validity: formal validity, plausibility, and predictive validity [22].

*Formal validity* assesses the degree to which correct methods are applied correctly. The model structure of DYNAMO-HIA is well-founded in epidemiological evidence – incidence, prevalence, and excess mortality – and demographic modeling practice, i.e. a multistate Markov-type model of chronic disease with explicit risk-factor states and inclusion of intermediate diseases.

*Plausibility* assesses the degree to which an observer deems the theoretical framework understandable, applicable, and plausible. Hence, DYNAMO-HIA deliberately restricts itself to the well-established causal chain “risk-factor exposure -> incidence ->prevalence -> disease-related mortality -> overall population health” and requires only data that is available in sufficient quality for the most common diseases (e.g. cancer, CVD, diabetes, COPD) and risk-factors (e.g. smoking, BMI, alcohol) in developed countries. In the Swedish application example, our results for the number of excess deaths is slightly lower than estimates based on a regression approach utilizing historical relationships and aggregate-data pooled from several Nordic countries [18]. One reason for this difference lies in the relative risks on all-cause mortality used in our illustration. These are taken from epidemiological studies and capture only the effect of individual exposure, i.e. drinking behavior. Consequently, our results do not account for broader effects that a change in alcohol consumption may have on population health, i.e. abstainers or moderate drinkers becoming victims of increased alcohol-induced violence or accidents caused by the increased number of intoxicated drinkers.

Plausibility and well-established formal methods should not be mistaken for constantly delivering expected results. Dynamic projections may reveal counterintuitive yet correct results and, hence, lead to important insights. In the smoking application, for example, the number of breast cancer cases in the never-smoker scenario is larger than in the reference scenario, although smoking has no causal link to breast cancer incidence. This seemingly unexpected result is caused by an increase in overall longevity of a healthier living population and, hence, an increased number of females susceptible to breast cancer. This phenomenon is well known among modelers of health care costs; dynamic analysis has shown repeatedly that a population-level reduction in obesity or smoking may lead to higher health care costs in the long run [23], [24].

*Predictive validity* is the degree to which predictions are confirmed by facts; according Veerman et al [22], however, this criterion usually cannot be established in the context of HIA. The sometimes decades-long time lag between a change in policy and a change in the corresponding health effects makes it difficult to conduct a full evaluation of the HIA prediction. Moreover, a HIA might influence policy in such a way as to (successfully) invalidate its own predictions.

We emphasize that a software model like DYNAMO-HIA is only a decision-support tool. It helps to quantify the expected differences in population health given two (or more) different scenarios: one of them a baseline scenario (without the intervention) and one (or more) scenario(s) with intervention(s). It does not predict the development of future population health as such. Decision makers must be constantly aware that real-world phenomena are necessarily more complex and that no model can predict future events with 100% accuracy. In HIA, it may be useful to avoid calling the results of mathematical models ‘predictions’, but rather *projections* of “what if ” scenarios in a clearly defined and simplifying framework. The term ‘prediction’ should be reserved for the entire process, in which a software model is only one element of the utilized evidence [9], [25], [26].

Internal validity was extensively tested. To allow future thorough checking of cross validity by outside experts as well, the software and the source code are publicly available (www.DYNAMO-HIA.eu). In its current form, DYNAMO-HIA also facilitates unproblematic one- and multi-way sensitivity analysis, by allowing easy manipulation of all input parameters. Like most other population health models, however, the current version of DYNAMO-HIA does not include a probabilistic sensitivity analysis (PSA). Implementing a PSA in population health models is time and cost intensive; the extra data needed to conduct a PSA are difficult to obtain and preparing them requires expert knowledge. However, DYNAMO-HIA can be used in batch mode, allowing users with sufficient computing skills to build a PSA shell around the software, if desired.

DYNAMO-HIA is available for free download and includes a data set covering a large number of EU countries (www.dynamo-hia.eu). This internally consistent data set has prevalence data for three risk-factors (smoking, BMI, alcohol), nine diseases (incidence, prevalence, excess mortality), and population data (e.g. total mortality, projected number of newborns). This data set allows instant use of DYNAMO-HIA for the covered countries. However, DYNAMO-HIA is also usable with external data on other countries, (sub-)populations, diseases, or risk-factors. Furthermore, the already included data set can be easily updated when more recent data become available. DYNAMO-HIA can be used for a range of applications, in particular if additional data are available.

Recent application of DYNAMO-HIA include comparison of tobacco control scenarios [27]; the effect of an increase in obesity levels for the Dutch population [13]; the EU-wide gains in population health when increasing prices on alcohol [28]; and the potential health gains and losses in the EU achievable through feasible prevalences of life-style related risk factors [29]. The current focus of DYNAM-HIA is on policies at the national level, but the software can, in principle, also be used for applications at the regional or local level.

### Conclusion

DYNAMO-HIA differs from other population health models for HIA [20] in several important aspects. From the outset, it has been designed for public use within HIA-applications by featuring a user-friendly graphic interface, and employing a model structure that ensures accurate simulation using epidemiological evidence while having modest data needs.

## Supporting Information

Table S1 Overview of data sources for disease data used in the example applications.
(DOCX)

Table S2 Overview of data sources for risk factors used in the example applications.
(DOCX)

Table S3 Overview of relative risks from alcohol to diseases and total mortality used in the example applications (below the age of 15 all relative risks are set 1).
(DOCX)

Table S4 Overview of relative risks from smoking to diseases and total mortality used in the example applications (below the age of 35 all relative risks are set to 1).
(DOCX)

Table S5 Overview of relative risks from diabetes to IHD and stroke used in the example applications.
(DOCX)
