# Supplementary material for: DYNAMO-HIA–A Dynamic Modeling Tool for Generic Health Impact Assessments
Source: PLoS One. 2012 May 10;7(5):e33317. doi: 10.1371/journal.pone.0033317 (PMC3349723; doi:10.1371/journal.pone.0033317)
Supplement: Table S1 — Overview of data sources for disease data used in the example applications. (DOCX) [file pone.0033317.s001.docx]

**Table S1: Overview of data sources for disease data used in the example applications**

|  |  | Sweden |  | UK |
| --- | --- | --- | --- | --- |
|  | *Prevalence/* | Back calculated using  DisMod II |  | Back calculated using  DisMod II |
| All five cancers included | *Incidence* | Cancer Incidence in 5 Continents. Vol IX , 100% of population (1998-2002)  (remission equal to zero) |  | Cancer Incidence in 5 Continents. Vol IX, Some 95% of population (periods vary by registry between 1998-2002)  (remission equal to zero) |
|  | *Excess Mortality/ Case Fatality* | WHO , mortality database, 100% of population (2000-2002) |  | WHO , mortality database, 100% of population (2000-2002) |
|  |  |  |  |  |
|  | *Prevalence* | Ostergotland Study |  | UK GPRD |
| Diabetes | *Incidence* | IPM based on prevalence & GPRD RR |  | IPM based on prevalence & GPRD RR |
|  | *Excess Mortality/ Case Fatality* | Based on RR from UK GPRD |  | UK GPRD |
|  |  |  |  |  |
|  | *Prevalence* | Back calculated using  DisMod II |  | Back calculated using  DisMod II |
| IHD | *Incidence* | Based UK GPRD incidence adjusted for Swedish IHD mortality |  | UK GPRD |
|  | *Prevalence* |  |  |  |
|  | *Excess Mortality/Case Fatality* | Based on RR from UK GPRD |  | UK GPRD |
|  |  |  |  |  |
|  | *Incidence* | Derived from smoking prevalence |  | UK GPRD |
| COPD | *Prevalence* | Back calculated using  DisMod II |  | UK GPRD |
|  | *Excess Mortality/ Case Fatality* | Based on RR from UK GPRD |  | UK GPRD |
|  |  |  |  |  |
|  | *Incidence* | WHO estimates Truelson et al review(2002) |  | WHO estimates Truelson et al review(2002) |
| Stroke | *Prevalence* | IPM based on incidence & GPRD RR |  | IPM based on incidence & GPRD RR |
|  | *Excess Mortality/ Case Fatality* | Based on RR from UK GPRD |  | UK GPRD |
| References:  http://www.who.int/healthinfo/global_burden_disease/ tools_software/en/  http://ci5.iarc.fr/CI5i-ix/ci5i-ix.htm  http://www.who.int/whosis/whosis/  www.gprd.com  Kruijshaar ME, Barendregt JJ, Hoeymans N. The use of models in the estimation of disease epidemiology. 2002; Bull World Health Organ. 80(8):622-8.  T. Truelsen/B. Piechowski-Jóźwiak/R. Bonita/C. Mathers/J. Bogousslavsky/G. Boysen, Stroke incidence and prevalence in Europe: a review of available data, in: European journal of neurology : the official journal of the European Federation of Neurological Societies 13 (2006) 6, 581–598.  Ann-Britt E. Wiréhn/H. Mikael Karlsson/John M. Carstensen, Estimating disease prevalence using a population-based administrative healthcare database, in: Scandinavian journal of public health 35 (2007) 4, 424–431.  Further details available on the data reports on www.dynamo-hia.eu | | | | |
