# Supplementary material for: DYNAMO-HIA–A Dynamic Modeling Tool for Generic Health Impact Assessments
Source: PLoS One. 2012 May 10;7(5):e33317. doi: 10.1371/journal.pone.0033317 (PMC3349723; doi:10.1371/journal.pone.0033317)
Supplement: Table S2 — Overview of data sources for risk factors used in the example applications. (DOCX) [file pone.0033317.s002.docx]

Table S2: Overview of data sources for risk factors used in the example applications

|  | Name of survey, year | Number of respondents | Age range of respondents |  |
| --- | --- | --- | --- | --- |
| Sweden (alcohol) | The (Alcohol) Monitoring Study, 2002 | N=~18,000 (national sample) | 16-80 years |  |
| UK (Smoking) | English Health Survey, 2001 | N=15767 (national sample for England, covering some 85% of the UK population) | 16 years and abour |  |
| References:  Leifman H, Gustafsson NK. En skål för det nya millenniet., SoRAD, 2003.  Johansson P, Jarl J, Eriksson A, Eriksson M, Gerdtham UG, Hemström Ö, Hradilova Selin K, Lenke L, Ramstedt M, Room R. The Social Costs of Alcohol in Sweden 2002, SoRAD, 2006.  Schaap et al. Specification of data files created within the EUROTHINE project. Harmonized files based on National Health Interview Surveys. Rotterdam: Erasmus MC; 2006.  Further details available on the data reports on www.dynamo-hia.eu. | | | | |
