# Supplementary material for: DYNAMO-HIA–A Dynamic Modeling Tool for Generic Health Impact Assessments
Source: PLoS One. 2012 May 10;7(5):e33317. doi: 10.1371/journal.pone.0033317 (PMC3349723; doi:10.1371/journal.pone.0033317)
Supplement: Table S3 — Overview of relative risks from alcohol to diseases and total mortality used in the example applications (below the age of 15 all relative risks are set 1). (DOCX) [file pone.0033317.s003.docx]

Table S3: Overview of relative risks from alcohol to diseases and total mortality used in the example applications (below the age of 15 all relative risks are set 1)

| Outcome | Males aged 15 years and over | | | | |  | Females aged 15 years and over | | | | |
| --- | --- | --- | --- | --- | --- | --- | --- | --- | --- | --- | --- |
|  | Drinking categories (grams per day) | | | | |  | Drinking categories (grams per day) | | | | |
|  | 0 - <0.25 | 0.25 - <20 | 20 - <40 | 40 - <60 | ≥60 |  | 0 - <0.25 | 0.25 - <20 | 20 - <40 | 40 - <60 | ≥60 |
| All-cause mortality |  |  |  |  |  |  |  |  |  |  |  |
| Persons Aged 16-24  Persons Aged 25-34  Persons Aged 35-44  Persons Aged 45-54  Persons Aged 55-64  Persons Aged 65-74  Persons Aged 75-84  Persons Aged 85-95 | 1.00  1.00  1.00  1.00  1.00  1.00  1.00  1.00 | 1.07  1.05  1.00  0.96  0.94  0.94  0.95  0.96 | 1.25  1.21  1.10  1.01  0.98  0.97  0.97  0.98 | 1.48  1.40  1.23  1.10  1.04  1.02  1.02  1.02 | 1.88  1.75  1.47  1.26  1.16  1.11  1.11  1.09 |  | 1.00  1.00  1.00  1.00  1.00  1.00  1.00  1.00 | 1.04  1.04  1.03  1.02  1.00  0.99  0.98  0.98 | 1.17  1.15  1.15  1.13  1.09  1.06  1.05  1.03 | 1.31  1.29  1.30  1.26  1.22  1.17  1.15  1.12 | 1.58  1.54  1.56  1.51  1.46  1.38  1.35  1.27 |
| IHD | 1.00 | 0.82 | 0.82 | 0.87 | 1.13 |  | 1.00 | 0.82 | 0.82 | 0.87 | 1.13 |
| Stroke | 1.00 | 0.91 | 1.01 | 1.18 | 1.55 |  | 1.00 | 0.7 | 0.79 | 1.08 | 2.74 |
| Diabetes mellitus | 1.00 | 0.72 | 0.86 | 1.00 | 1.00 |  | 1.00 | 0.72 | 0.86 | 1.00 | 1.00 |
| COPD | 1.00 | 1.00 | 1.00 | 1.00 | 1.00 |  | 1.00 | 1.00 | 1.00 | 1.00 | 1.00 |
| Lung cancer | 1.00 | 1.00 | 1.00 | 1.00 | 1.00 |  | 1.00 | 1.00 | 1.00 | 1.00 | 1.00 |
| Colon cancer | 1.00 | 1.00 | 1.08 | 1.30 | 1.72 |  | 1.00 | 1.00 | 1.11 | 1.33 | 1.62 |
| Oral cancer | 1.00 | 1.31 | 2.08 | 3.02 | 4.32 |  | 1.00 | 1.33 | 2.18 | 3.26 | 4.85 |
| Breast cancer | 1.00 | 1.00 | 1.00 | 1.00 | 1.00 |  | 1.00 | 1.00 | 1.23 | 1.42 | 1.68 |
| Esophageal cancer | 1.00 | 1.17 | 1.61 | 2.19 | 3.18 |  | 1.00 | 1.17 | 1.61 | 2.19 | 3.18 |
| Greene CC, Bradley KA, Bryson CL et al. The association between alcohol consumption and risk of COPD exacerbation in a veteran population. Chest 2008;134:761-767.  International Agency for Research on Cancer (IARC). Meeting summary: Volume 96: Alcoholic Beverage Consumption and Ethyl Carbamate (Urethane) 6-13 February 2007. Lyon: IARC, 2007.  Rehm J, Sulkowska U, Mańczuk M, Boffetta P, Powles J, Popova S, Zatoński W. Alcohol accounts for a high proportion of premature mortality in central and eastern Europe. Int J Epidemiol. 2007 Apr;36(2):458-67. Epub 2007 Jan 24.  Tabak C, Smit HA, Räsänen L, Fidanza F, Menotti A, Nissinen A, Feskens EJ, Heederik D, Kromhout D. : Alcohol consumption in relation to 20-year COPD mortality and pulmonary function in middle-aged men from three European countries. Epidemiology. 2001; 12:239-245.  White IR, Altmann DR, Nanchahal K. ‘Optimal’ levels of alcohol consumption for men and women at different ages, and the all-cause mortality attributable to drinking. London: London School of Hygiene and Tropical Medicine, 2000.[Technical Report]  White IR, Altmann DR, Nanchahal K. Alcohol consumption and mortality: modelling risks for men and women at different ages. British Medical Journal 2002; 325:191-194.  World Cancer Research Fund / American Institute for Cancer Research. Expert Report, Food, Nutrition, Physical Activity and the Prevention of Cancer: a Global Perspective. Washington DC: AICR, 2007. | | | | | | | | | | | |
| Further details available on the data reports on www.dynamo-hia.eu | | | | | | | | | | | |
