# Supplementary material for: DYNAMO-HIA–A Dynamic Modeling Tool for Generic Health Impact Assessments
Source: PLoS One. 2012 May 10;7(5):e33317. doi: 10.1371/journal.pone.0033317 (PMC3349723; doi:10.1371/journal.pone.0033317)
Supplement: Table S4 — Overview of relative risks from smoking to diseases and total mortality used in the example applications (below the age of 35 all relative risks are set to 1). (DOCX) [file pone.0033317.s004.docx]

Table S4: Overview of relative risks from smoking to diseases and total mortality used in the example applications (below the age of 35 all relative risks are set to 1)

| Outcome | Male aged 35 and above | | |  | Female aged 35 and above | | |
| --- | --- | --- | --- | --- | --- | --- | --- |
|  | Never Smoker | Current Smoker | Former Smoker |  | Never Smoker | Current Smoker | Former Smoker |
| All-cause mortality |  |  |  |  |  |  |  |
| Persons Aged 35–39    Persons Aged 40-44    Persons Aged 45–49    Persons Aged 50–54    Persons Aged 55–59    Persons Aged 60-64    Persons Aged 65+ | 1.00  1.00  1.00  1.00  1.00  1.00  1.00 | 2.07  2.07  2.07  2.07  2.07  2.07  2.07 | 1.35  1.35  1.35  1.35  1.35  1.35  1.35 |  | 1.00  1.00  1.00  1.00  1.00  1.00  1.00 | 1.74  1.74  1.74  1.74  1.74  1.74  1.74 | 1.23  1.23  1.23  1.23  1.23  1.23  1.23 |
| Lip, Oral Cavity, Pharynx |  |  |  |  |  |  |  |
| Persons Aged 35–39    Persons Aged 40-44    Persons Aged 45–49    Persons Aged 50–54    Persons Aged 55–59    Persons Aged 60-64    Persons Aged 65+ | 1.00  1.00  1.00  1.00  1.00  1.00  1.00 | 10.89  10.89  10.89  10.89  10.89  10.89  10.89 | 3.40  3.40  3.40  3.40  3.40  3.40  3.40 |  | 1.00  1.00  1.00  1.00  1.00  1.00  1.00 | 5.08  5.08  5.08  5.08  5.08  5.08  5.08 | 2.29  2.29  2.29  2.29  2.29  2.29  2.29 |
| Esophagus |  |  |  |  |  |  |  |
| Persons Aged 35–39    Persons Aged 40-44    Persons Aged 45–49    Persons Aged 50–54    Persons Aged 55–59    Persons Aged 60-64    Persons Aged 65+ | 1.00  1.00  1.00  1.00  1.00  1.00  1.00 | 6.76  6.76  6.76  6.76  6.76  6.76  6.76 | 4.46  4.46  4.46  4.46  4.46  4.46  4.46 |  | 1.00  1.00  1.00  1.00  1.00  1.00  1.00 | 7.75  7.75  7.75  7.75  7.75  7.75  7.75 | 2.79  2.79  2.79  2.79  2.79  2.79  2.79 |
| Lung cancer |  |  |  |  |  |  |  |
| Persons Aged 35–39    Persons Aged 40-44    Persons Aged 45–49    Persons Aged 50–54    Persons Aged 55–59    Persons Aged 60-64    Persons Aged 65+ | 1.00  1.00  1.00  1.00  1.00  1.00  1.00 | 1.30  1.00  5.78  24.97  34.02  31.47  28.40 | 1.00  1.00  2.37  10.70  11.66  11.71  9.70 |  | 1.00  1.00  1.00  1.00  1.00  1.00  1.00 | 2.00  1.00  18.08  11.14  17.87  13.32  17.49 | 1.00  1.00  8.07  3.28  5.33  4.91  5.54 |
| IHD |  |  |  |  |  |  |  |
| Persons Aged 35–39    Persons Aged 40-44    Persons Aged 45–49    Persons Aged 50–54    Persons Aged 55–59    Persons Aged 60-64    Persons Aged 65+ | 1.00  1.00  1.00  1.00  1.00  1.00  1.00 | 3.25  4.71  5.85  3.69  2.71  2.39  1.91 | 1.21  1.15  2.03  1.93  1.64  1.58  1.40 |  | 1.00  1.00  1.00  1.00  1.00  1.00  1.00 | 1.00  1.89  7.71  5.69  3.06  2.56  2.48 | 1.44  2.25  2.08  2.95  1.19  1.08  1.22 |
| Stroke |  |  |  |  |  |  |  |
| Persons Aged 35–39    Persons Aged 40-44    Persons Aged 45–49    Persons Aged 50–54    Persons Aged 55–59    Persons Aged 60-64    Persons Aged 65+ | 1.00  1.00  1.00  1.00  1.00  1.00  1.00 | 1.00  1.05  3.75  6.08  3.96  2.55  2.69 | 1.00  1.00  1.00  2.24  1.14  1.01  1.29 |  | 1.00  1.00  1.00  1.00  1.00  1.00  1.00 | 2.00  5.67  8.22  4.58  5.77  2.76  2.58 | 1.00  2.25  1.19  1.38  1.22  1.28  1.14 |
| COPD |  |  |  |  |  |  |  |
| Persons Aged 35–39    Persons Aged 40-44    Persons Aged 45–49    Persons Aged 50–54    Persons Aged 55–59    Persons Aged 60-64    Persons Aged 65+ | 1.00  1.00  1.00  1.00  1.00  1.00  1.00 | 1.00  1.00  1.00  8.13  9.80  13.21  18.93 | 1.00  1.00  1.00  3.06  8.25  12.65  11.92 |  | 1.00  1.00  1.00  1.00  1.00  1.00  1.00 | 1.00  1.00  1.00  12.92  9.47  11.19  14.72 | 1.00  1.00  1.00  7.39  5.55  6.63  9.73 |
| References:  Ellison LF et al. Health consequences of smoking among Canadian smokers: An update. Chronic Dis Can 1999; 20:36-9.  American Cancer Society´s Cancer Prevention Study II age-specific relative risks (1982-1988).  American Cancer Society´s Cancer Prevention Study II age-specific relative risks (1982-1988).  Tanuseputro P, Manuel DG, Schultz SE, Johansen H, Mustard CA. Improving population attributable fraction methods: examining smoking-attributable mortality for 87 geographic regions in Canada. Am J Epidemiol. 2005 Apr 15;161(8):787-98 | | | | | | | |
| Further details available on the data reports on www.dynamo-hia.eu | | | | | | | |
