# Supplementary material for: DYNAMO-HIA–A Dynamic Modeling Tool for Generic Health Impact Assessments
Source: PLoS One. 2012 May 10;7(5):e33317. doi: 10.1371/journal.pone.0033317 (PMC3349723; doi:10.1371/journal.pone.0033317)
Supplement: Table S5 — Overview of relative risks from diabetes to IHD and stroke used in the example applications. (DOCX) [file pone.0033317.s005.docx]

Table S5: Overview of relative risks from diabetes to IHD and stroke used in the example applications

|  | Males | Females |
| --- | --- | --- |
| Diabetes to IHD |  |  |
| Persons Aged up to 55    Persons Aged 56+ | 2.66  1.93 | 3.53  2.59 |
|  |  |  |
| Diabetes to stroke |  |  |
| Persons Aged up to 49    Persons Aged 50+ | 2.00  1.80 | 2.90  2.20 |
| Yusuf S, Hawken S et al. Effect of potentially modifiable risk factors associated with myocardial infarction in 52 countries (the INTERHEART study): case-control study. 2004; 364: 937- 52.  Barrett-Connor E, Khaw KT. Diabetes mellitus: an independent risk factor for stroke? Am J Epidemiol. 1988 Jul;128(1):116-23. Gu K, Cowie CC, Harris MI. Mortality in adults with and without diabetes in a national cohort of the U.S. population, 1971-1993. Diabetes Care. 1998Jul;21(7):1138-45. | | |
| Further details available on the data reports on www.dynamo-hia.eu | | |
